# Aligning clinical research ethics with community-engaged and participatory research in the United States

**DOI:** 10.3389/fpubh.2023.1122479

**Published:** 2023-05-04

**Authors:** Milton (Mickey) Eder

**Affiliations:** Department of Family Medicine and Community Health, University of Minnesota Medical School, Minneapolis, MN, United States

**Keywords:** clinical research, ethics, community, community engaged, participatory, community-based participatory research, institutional review board, review

## Abstract

The professional role in ethical review of research in which boards review proposed research involving human beings continues to evolve. The scholarly literature on institutional review boards in academic centers of the United States, at which a majority of the community engaged and participatory research emanates and is reviewed, suggests the need to implement changes in board education, the infrastructure supporting review, and the accountability of review. The recommendations for change advanced in this perspective involve enhancing reviewer knowledge of local community contexts and developing an infrastructure that supports engagement in and dialogue among individuals involved in community-academic research to inform ethical review and the assessment of review outcomes. Additionally, recommendations regarding putting an institutional infrastructure in place are advanced in order to sustain community engaged and participatory research. The infrastructure can also support the collection and review of outcome data as the foundation of accountability. The recommendations outlined intend to improve clinical research ethics reviews of community-engaged and participatory research.

## Introduction

Throughout the past century, professional voices have predominated in articulating, interpreting and applying ethical principles in the review of research involving human beings (1–4). Eleven individuals with expertise in the medical and behavioral sciences, ethics, law and public policy produced the Belmont Report, articulating basic ethical principles for the prospective review of research participant protection in terms of safety and rights (5). The Report furthered reliance on the review of research by independent boards (6), which have proliferated with increases in funding and in the number of research studies (7–10). Private or for-profit Institutional Review Boards arose to meet the demand for review (11), serving researchers without institutional affiliation and institutions seeking to comply with conflict of interest policies. Associations of professionals have also shaped the management of boards and review processes (12–14).

The growth in the number of research studies and reviews has been accompanied by the emergence of new research methods and study designs. PubMed citations show “pragmatic clinical trial/trials” publications increasing from an average of three per year (1984–2012) to over 200 average citations annually for the last decade. Similarly, “comparative effectiveness trial/trials” citations begin to markedly increase around 2010. Increases in the number of community-based participatory research and community-engaged research studies and publications started to occur even earlier. These types of studies comprise subsets of research conducted within community settings and with community partners (15). The proliferation and diversification in research studies and settings present numerous and sometimes unrecognized challenges for ethical board review.

## What has ethical review of clinical research looked like?

Few studies have closely examined the structure and function of the institutional review board (IRB): We know even less about private or for-profit IRBs (16). Structure has typically been interpreted as board composition, which forms a foundation for examining board function and board member interaction.

Researchers examining board composition often focus on the requirement of boards to include a non-scientist and an individual unaffiliated with an IRB’s institutional sponsor. While the same individual can fulfill both roles and sometimes does, research into board composition often combines these two roles. The 28 “non-scientists” serving on the fourteen IRBs at the National Institute of Health reported actively contributing to board decisions and feeling they were listened to by others on the board. A large majority felt a primary responsibility for reviewing the informed consent documents (17). Studies of nonaffiliated and non-scientist IRB members within academic health centers reported members in these roles feeling ill prepared to actively contribute to board discussions and not respected (18, 19). All board members reported uncertainty about the roles of non-affiliated and non-scientist board members (20).

A 2011 systematic review found 43 studies of US academic IRBs reporting empirical evidence about board “structure, process, outcomes, effectiveness, or review variation (21).” Collectively IRB interpretation and application of federal guidance to protocols varied. Additionally, the review noted an absence of evidence about the quality of reviews and about IRB effectiveness in protecting human research participants. An absence of data regarding IRB quality and effectiveness of their research reviews persists.

A subsequent study explained variation in board review by summarizing research findings about risk assessment and decision-making at both the individual and group levels. Variation could result from how an individual responds to the wording used to describe risk, from the degree of familiarity with a procedure or sources of stigma as well as from trust in the people involved. Culture and political orientation can also be sources of variation in assessments of risk and its severity. Patterns in the perception and responses to risk were outlined to raise awareness about sources of bias and potentially improve review consistency. However, variation could legitimately result from a knowledge of local context (22).

A qualitative study of a single IRB characterized board members deferring to the professionals with expertise in the room (23). A second study of board structure and function, which was focused on review of social, behavioral, and economic research protocols, found IRBs largely populated by individuals possessing medical expertise and experience reviewing clinical trials. Observation, again of one IRB, suggested heightened scrutiny of social, behavioral, and economic research, including minimal risk projects. Field notes reported more board members actively participated in social behavioral reviews in comparison to biomedical protocols (24). The authors posited that board members reviewing social and behavioral protocols felt empowered to assume a sense of their own expertise. They recommended increasing the proportion of board members with social science expertise and adding members to represent research participants (25). They also recommended board education related to social and behavioral research.

Decades of growth in clinical research has fueled concern about IRB workload and mission creep. Workloads may be lessened somewhat by using reliance agreements to minimize the number of boards that review a protocol. Mission creep is more complicated. It may arise in traditional hospital-based clinical trials due to the current emphasis on increasing heterogeneity among research participants, which may lead boards to consider social/community, economic, environmental and cultural contexts to address issues of autonomy, risk and benefit and social justice (4): As suggested above, reviews might vary due to local contexts and perhaps when factoring in participants from populations that do and do not experience health disparities. Should boards scrutinize proposed samples for their representativeness and recruitment plans in considerations of social justice and equity? Mission creep is certainly a concern for academic health center IRBs where the majority of community-engaged and participatory research reviews likely occur.

The formation of IRBs focused on social and behavioral research seems one response to issues of board composition and mission creep. However, distinguishing biomedical from social and behavioral research is unlikely to ever prove adequate for the exploration of ethical issues that arise from conducting research among diverse populations in community settings, using methods and designs common among community-placed, community-engaged, comparative effectiveness, community-based participatory, participatory action, translational, implementation and dissemination, research studies.

## Expanding ethical review

There are limited examples of directly engaging community perspectives in considerations of research ethics. The University of California-San Francisco (UCSF) assembled individuals from local communities who were already working with UCSF researchers and who possessed research expertise to bring together community voices within the ethical review of the institution’s COVID-19 related protocols (26).

Two decades earlier, the Morehouse School of Medicine Prevention Research Center Community Coalition Board articulated principles and values for the community review of research. Their principles and values included mutual respect and justice for all people, a commitment to the principle of self-determination, and a recognition that structures and systems within which individuals live, work, and play, limit individual autonomy. Contrary to guidance instructing IRBs to avoid interpreting the creation of community jobs or clinical infrastructure as a research derived benefit (27), the Community Coalition Board required projects to demonstrate a contribution to the community capacity to benefit from research processes and outcomes (28).

The Bronx Community Research Review Board, a product of The Bronx Health Link and the Albert Einstein College of Medicine, was formed to provide consultation about “community-based research proposals.” Bronx residents also demonstrated “substantial interest” in understanding how proposals responded to local needs. The Community Research Review Board goals included community education about clinical research conduct, ethics and the research occurring in the community. It expressly recognized it was not an IRB but it still sought to empower community voices through consultation with researchers and by maximizing benefits of conducting research, of implementation and advocacy based on findings. Community member training for Board service was grounded in Paulo Freire’s conceptualization of participation and community empowerment by expecting trainees to reflect on and further develop the training curriculum (29).

Communities and institutions developed other approaches to research consultation (30). The Community Engagement Studio has gained prominence as a reliable way to obtain community member input on research projects, particularly recruitment and retention plans and materials (31, 32). Academic researchers have proposed sharing information about community consultations to expand their understanding of research ethics for projects collaboratively conducted with community partners (33).

A conceptual model recommending the establishment of an independent community ethical review board, positioned between IRBs and community advisory boards, has been put forward (34). A recent Patient Centered Outcomes Research Institute (PCORI) study similarly recommended sustaining engagement and partner relationships and also encouraging team science by supporting community member and stakeholder participation on research teams (35). The shift from managing research projects individually to an infrastructure for the ongoing management of community relationships and partnerships would begin to address the marginalization of community member voices on IRBs and enhance the potential for local community voices to contribute to the exploration and application of research ethics for community-engaged and participatory projects (36–41).

## Recommendations: research ethics and community

Prior comments pointed to a professional hegemony in the review of research protocols and in the application of ethical guidance within academic medical center research. Examples were also provided of community initiatives seeking to understand the value of research for communities and expressing expectations of direct involvement in the research occurring within their communities. Also mentioned was literature about research review issues with a focus on challenges faced by researchers involved in community engaged and participatory research. What is lacking in the literature is empirical evidence about the quality and outcomes of IRB decisions. The failure to evaluate and critically reflect on review determinations and their outcomes creates a gap in IRB accountability. It fails to address community mistrust generated by a history of research abuses, it also fails to confirm that the ethical norms developed to guide research that involves human beings within academic clinical contexts are appropriate to or appropriately interpreted and applied to research conducted in community contexts (42).

While not absolute, the distinction between research contexts is not trivial as is evident in Woolf’s contrast between two research stages within translational science (43). The two stages present a contrast in research designs and purposes. Stage one study designs are used to obtain data about the efficacy of new clinical therapies, while stage two designs attend to their effectiveness. Closed system designs ideally control for a single variable to demonstrate causality. By contrast, open system designs acknowledge variability across multiple real-world settings, producing data to address the generalizability of therapies (e.g., pragmatic and comparative effectiveness trials). These contrasting study designs and their focus on establishing internal (closed system) and external (open system) validity, complicate ethical considerations regarding informed consent (e.g., SUPPORT Trial) (44), assessments of risks and benefits (45) and social justice. We begin to address concerns about research ethics by advancing recommendations to empower community perspectives and participation within the education of IRB members, institutional infrastructure, and review board accountability.

### Education or awareness raising

The Collaborative Institutional Training Initiative (CITI) which provides online training courses for both researchers and IRB members developed a course that introduces community-based participatory research and community-engaged research approaches and ethical issues. While a recognition of increased research activity, this general introduction should be augmented within the ongoing education provided board members by their institutions (46). Continuing education for IRB members might introduce the institution’s approach to community within its Community Health Needs Assessment or provide board members with information about the diverse populations within the catchment area, improving board member understanding of community health issues and outcomes (47–51). Board education could explore collaborative, participatory, and qualitative research designs and methods [e.g., photovoice (52, 53)], team science (54), partnership assessment (interpersonal and research) (55), health literacy and information design (56). While such educational efforts may not in itself overcome the limited community expertise on boards, it could improve board member understanding of specific community contexts within which the community engaged and participatory research they are reviewing will occur.

### Building infrastructure

An institutional infrastructure to sustain bi-directional community-academic dialogue and involvement in decision-making should be capable of supporting community partnerships and service learning and of contributing to community health needs assessments and project and program evaluation (57, 58). A standing committee betwixt and between IRBs and research project advisory boards has been proposed to enable colloquial voices to intervene in professional discourse regarding the policies, practices and norms of community engaged and participatory clinical research (59). A standing group could also be a resource of individuals from the community to participate in assessing community-based research conduct, which would involve conducting assessments as is recommended below. The individuals could also help disseminate messages to diverse communities about research (e.g., the relevance of specific projects to community health; the importance of research involving individuals from the community to inform evidence-based medicine) (56). Such a group could also help to situate research along the blurred boundary between research and clinical care within learning health systems.

Academic institutions with standing community advisory groups could add research ethics as a recurring item to their meeting agendas. Members from different community advisory boards could be brought together, providing a counterbalance to the fragmentation produced by project specific advisory groups. The group could include non-affiliated IRB members at the institution. Institutions with multiple IRBs could constitute a group from their non-affiliated and non-scientist board members. Depending on the responsibilities accorded the group, it could meet a few times a year and involve minimal cost to the institution. A cost benefit analysis could be conducted to consider whether the infrastructural cost increases the institution’s negotiated indirect rate. The analysis of cost and benefit should also consider whether the increased attention to the ethics of community engaged and participatory research is associated with an increase in funded projects as well as engendering trust within the community that facilitates research participation. While the cost would depend on the form and responsibilities of the group, the purpose remains to increase the engagement of community perspectives in determining what constitutes ethical research conduct, particularly for research conducted through community partnerships and within community contexts (60).

The group could also include community-based clinicians. Why this suggestion may seem to reinforce the hegemony of professionals in determining research ethics, community-based clinicians are not typically research professionals (61); they possess different expertise, whose value has been demonstrated in determining local standards of clinical care (62, 63). The involvement of primary care clinicians would also be an asset with the expanding integration of research into community care contexts. Adding representatives from primary care could inform discussions of minimizing potential risks and maximizing potential benefits for clinical trial, implementation and dissemination research. The Hispanic Chronic Renal Insufficiency Cohort study conducted in Chicago offers one example (64, 65). Local study initiation efforts included the lead researcher (i.e., Principal Investigator) visiting primary care clinics and Federally Qualified Health Centers to explain this 5-year prospective observational study to community clinicians. The lead researcher agreed to serve community clinicians as a resource for interpreting clinical data returned to research participants and developing patient care plans. This arrangement held within it a potential for benefit to individual participants and for the community through access to a clinical specialist with expertise not readily accessible in safety-net care contexts. While the H-CRIC arrangement was informal and more than a decade ago, engaging community clinicians can strengthen community partnerships and collaborations seeking to develop ways to generate collective benefit and pursue social justice. The regular engagement of community voices and discussion of community perspectives regarding the ethical conduct of research has the potential to improve the ethical oversight of research and further demonstrate university and academic health center commitments to partnership with communities.

### Accountability

IRB education and the organizational infrastructure to support research conducted in community contexts should both inform and be informed by assessments of review outcomes, particularly research team member-participant interactional outcomes and assessments of actual risks and benefits. Presently, however, there is little to no published data to assess the outcomes of board reviews. While we possess evidence of therapeutic misconception in which individuals conflate research with treatment, there is little to no published data regarding how well informed consent materials and processes contribute to an individual’s understanding of a specific research study. We know little to nothing about whether the payment offered participants is potentially coercive or whether what is offered is in any way consistent across comparable studies at an institution. Data is also lacking regarding participant and community experience of research participation. While institutions support human research protection programs and the IRBs who provide ethical review of proposed research and while accreditation indicates they are doing so successfully, there is scant empirical evidence to demonstrate that the ethical training of researchers and IRB review determinations are being translated into responsible ethical conduct (66, 67). While the responsibilities for conducting research ethically are clear, the lack of available data contributes to a gap in institutional accountability.

In order to demonstrate accountability, institutions should demonstrate that their review processes are generating the expected outcomes (68, 69). Institutions might start with interactions among IRB members by inquiring whether non-affiliated and non-scientist board members actually participate in board reviews and whether they feel listened to and respected. Put simply, does the board review process actually involve contributions from all required participants. Assessments of institutional review board performance could test approved informed consent documents to determine if they are meeting announced readability standards (70, 71). Readability can be easily examined through free, online utilities (e.g., https://www.online-utility.org/english/readability_test_and_improve.jsp) and might start by examining specific sections of what are often documents of 20 pages or more; it might prove useful to begin with the templated language that institutions require their researchers use to explain research or how the language used (e.g., to describe risks and benefits) may influence decision-making (72, 73). When it comes to the review process and informed consent, researchers have demonstrated that IRBs have regularly failed to demonstrate integrity by holding themselves accountable for meeting stated readability standards.

With the heightened awareness of the scientific importance of diversity in research participation in order to obtain evidence representative of the overall population, institutions could hold themselves accountable by comparing diversity of research recruitment and participation across minimal risk and also across more than minimal risk studies; they could compare participation in hospital-based clinical trials and trials conducted in community contexts. There are numerous potential comparisons that could help institutions assess research enrollments and inclusivity over time.

In addition to the recommendation to expand education for IRB members (74), institutions should review the information that researchers are required to provide for review. Again, while the data is extremely limited, it appears that IRB members may not have the necessary information about community partnerships and about the capacity and experience of community partners to support a research protocol available to conduct a thorough review of community engaged and participatory research (75, 76). There are numerous areas for institutional self-improvement regarding the review and oversight provided community-engaged and participatory research that would indicate a commitment to IRB’s primary responsibility of protecting research participants by minimizing their exposure to risk and supporting the production of benefit by every means possible.

## In closing

This overly brief review of research ethics for community-engaged and participatory research has overlooked stand-alone community ethical review practices (e.g., sovereign tribal nations) (77, 78). This limitation is not meant to minimize their importance nor dismiss their practices, but rather to acknowledge differences in legal status, particularly the continuities and discontinuities of individual and group identities. We acknowledge that cultural and linguistic differences add epistemological challenges for overcoming professional perspectives on late-stage clinical research, something which has been looked at extensively by Canadian researchers. Such challenges highlight assumptions about the universality of the autonomous individual while recognizing continuities and discontinuities within sociological or psychosocial conceptualizations of the person (79, 80).

The application of ethical principles and the review of research involving human beings must continue to evolve by conducting dialogues that collaboratively explore ethics and their axiological interpretations within clinical and health research. As suggested by the Association of American Medical Colleges, institutions capable of supporting sustained community-academic partnerships and disseminating information about those partnerships within diverse community contexts are more likely to become trustworthy community partners (81).

## Data availability statement

The original contributions presented in the study are included in the article/supplementary material, further inquiries can be directed to the corresponding author.

## Author contributions

The author confirms being the sole contributor of this work and has approved it for publication.

## Funding

Funding was provided by the Department of Family Medicine and Community Health, University of Minnesota Medical School.

## Conflict of interest

The author declares that the research was conducted in the absence of any commercial or financial relationships that could be construed as a potential conflict of interest.

## Publisher’s note

All claims expressed in this article are solely those of the authors and do not necessarily represent those of their affiliated organizations, or those of the publisher, the editors and the reviewers. Any product that may be evaluated in this article, or claim that may be made by its manufacturer, is not guaranteed or endorsed by the publisher.

## References

[1] Jonas H. Philosophical reflections on experimenting with human subjects. Daedalus. (1969) 98:219–47.

[2] Resnik DB. The ethics of research with human subjects: protecting people, advancing science, promoting trust *vol. 74*, 1st edn. USA: Springer (2018).

[3] McCarthy C. The origins and policies that govern institutional review boards In: Emanuel E Grady C Crouch R Lie R Miller F Wendler D, editors. The oxford textbook of clinical research ethics. New York, NY: Oxford University Press (2008). 541–50.

[4] Levine RJ. Research ethics committees. Encyclopedia of Bioethics (1995), Available at: https://www.encyclopedia.com/science/encyclopedias-almanacs-transcripts-and-maps/research-ethics-committees (Accessed September 28, 2022).

[5] National Commission for the Protection of Human Subjects of Biomedical and Behavioral Research. The Belmont Report: Ethical Principles and Guidelines for the Protection of Human Subjects of Research. Bethesda, MD: The Commission (1978) Available at: https://www.hhs.gov/ohrp/sites/default/files/the-belmont-report-508c_FINAL.pdf. 25951677

[6] Macklin R. How independent are IRBs? IRB. (2008) 30:15–9. PMID: 18814441

[7] Sekar K. National Institutes of Health (NIH) Funding: FY1996-FY2022. Updated June 29, 2021. Congressional Research Service R43341, (2021). Available at: https://sgp.fas.org/crs/misc/R43341.pdf (Accessed October 5, 2022).

[8] World Health Organization, Number of clinical trial registrations by location, disease, phase of development, age and sex of trial participants (1999-2021). Available at: https://www.who.int/observatories/global-observatory-on-health-research-and-development/monitoring/number-of-trial-registrations-by-year-location-disease-and-phase-of-development (Accessed October 5, 2022).

[9] ClinicalTrials.gov Trends, Charts, and Maps. Available at: https://clinicaltrials.gov/ct2/resources/trends (Accessed October 5, 2022).

[10] Matej M. Total number of registered clinical studies worldwide since 2000 (as of March 2022). Statista (2022). Available at: https://www.statista.com/statistics/732997/number-of-registered-clinical-studies-worldwide/ (Accessed October 5, 2022).

[11] Heath E. The noninstitutional review board: what distinguishes us from them? IRB. (1998) 20:8–11. doi: 10.2307/3563733, PMID: 11657585

[12] Public Responsibility in Medicine and Research. Available at: https://primr.org/ (Accessed November 23, 2022).

[13] The Association of Clinical Research Professionals. Available at: https://acrpnet.org/ (Accessed November 23, 2022).

[14] Society of Clinical Research Associates. Available at: https://www.socra.org/ (Accessed November 23, 2022).

[15] Ortiz K Nash J Shea L Oetzel J Garoutte J Sanchez-Youngman S. Partnerships, processes, and outcomes: a health equity-focused scoping meta-review of community-engaged scholarship. Annu Rev Public Health. (2020) 41:177–99. doi: 10.1146/annurev-publhealth-040119-094220, PMID: 31922931 PMC8095013

[16] Elliott C. White Coat Black Hat: Adventures on the Dark Side of Medicine. Boston, MA: Beacon Press (2010).

[17] Allison RD Abbott LJ Wichman A. Nonscientist IRB members at the NIH. IRB. (2008) 30:8–13. PMID: 18953771 PMC2919835

[18] Sengupta S Lo B. The roles and experiences of nonaffiliated and non-scientist members of institutional review boards. Acad Med J Assoc Am Med Coll. (2003) 78:212–8. doi: 10.1097/00001888-200302000-00019, PMID: 12584103

[19] Anderson EE. A qualitative study of non-affiliated, non-scientist institutional review board members. Account Res. (2006) 13:135–55. doi: 10.1080/08989620600654027, PMID: 16827216

[20] Klitzman R. Institutional review board community members: who are they, what do they do, and whom do they represent? Acad Med J Assoc Am Med Coll. (2012) 87:975–81. doi: 10.1097/ACM.0b013e3182578b54, PMID: 22622206 PMC3549463

[21] Abbott L Grady C. A systematic review of the empirical literature evaluating IRBs: what we know and what we still need to learn. J Emp Res Hum Res Ethics. (2011) 6:3–19. doi: 10.1525/jer.2011.6.1.3, PMID: 21460582 PMC3235475

[22] Pritchard IA. How do IRB members make decisions? A review and research agenda. J Empir Res Hum Res Ethics. (2011) 6:31–46. doi: 10.1525/jer.2011.6.2, PMID: 21680975

[23] Stark L. Behind Closed Doors: IRBs and the Making of Ethical Research (Morality and Society Series). Chicago, IL: University of Chicago Press (2012).

[24] De Vries R DeBruin DA Goodgame A. Ethics review of social, behavioral, and economic research: where should we go from here? Ethics Behav. (2004) 14:351–68. doi: 10.1207/s15327019eb1404_6, PMID: 16625729

[25] Robertson JA. Ten ways to improve IRBs. Hastings Cent Rep. (1979) 9:29–33. doi: 10.2307/3561699, PMID: 429060

[26] Eder MM Millay TA Cottler LB PACER Group. A compendium of community engagement responses to the COVID-19 pandemic. J Clin Transl Sci. (2021) 5:e133. doi: 10.1017/cts.2021.800, PMID: 34367677 PMC8326670

[27] Ross LF Loup A Nelson RM Botkin JR Kost R Smith GR. Nine key functions for a human subjects protection program for community-engaged research: points to consider. J Emp Res Hum Res Ethics: JERHRE. (2010) 5:33–47. doi: 10.1525/jer.2010.5.1.33, PMID: 20235862 PMC2963647

[28] Blumenthal DS. A community coalition board creates a set of values for community-based research. Prev Chronic Dis. (2006) 3:A16. Available at: http://www.cdc.gov/pcd/issues/2006/jan/05_0068.htm PMID: 16356369 PMC1500962

[29] Martin del Campo F Casado J Spencer P Strelnick H. The development of the Bronx community research review board: a pilot feasibility project for a model of community consultation. Prog Comm Health Partnersh. (2013) 7:341–52. doi: 10.1353/cpr.2013.0037, PMID: 24056516 PMC4795445

[30] Taylor HA Kass NE. Our two cents: research ethics consultation at Johns Hopkins Bloomberg School of Public Health. Am J Bioeth. (2008) 8:33–5. doi: 10.1080/15265160802109405, PMID: 18570099 PMC3110661

[31] Joosten YA Israel TL Head A Vaughn Y Villalta GV Mouton CP. Enhancing translational researchers' ability to collaborate with community stakeholders: lessons from the community engagement studio. J Clin Transl Sci. (2018) 2:201–7. doi: 10.1017/cts.2018.323, PMID: 30820357 PMC6382358

[32] Joosten YA Israel TL Williams NA Boone LR Schlundt DG Mouton CP. Community engagement studios: a structured approach to obtaining meaningful input from stakeholders to inform research. Acad Med J Assoc Am Med Coll. (2015) 90:1646–50. doi: 10.1097/ACM.0000000000000794, PMID: 26107879 PMC4654264

[33] Cho MK Taylor H McCormick JB Anderson N Barnard D Boyle MB. Building a central repository for research ethics consultation data: a proposal for a standard data collection tool. Clin Transl Sci. (2015) 8:376–87. doi: 10.1111/cts.12268, PMID: 25758372 PMC4888071

[34] Watkins BX Shepard PM Corbin-Mark CD. Completing the circle: a model for effective community review of environmental health research. Am J Public Health. (2009) 99 Suppl 3:S567–77. doi: 10.2105/AJPH.2008.149369, PMID: 19890159 PMC2774186

[35] Heckert A Forsythe LP Carman KL Frank L Hemphill R Elstad EA. Researchers, patients, and other stakeholders’ perspectives on challenges to and strategies for engagement. Res Involv Engagem. (2020) 6:60–18. doi: 10.1186/s40900-020-00227-0, PMID: 33042576 PMC7539495

[36] Garrett SB Dohan D Koenig BA. Linking broad consent to biobank governance: support from a deliberative public engagement in California. Am J Bioeth. (2015) 15:56–7. doi: 10.1080/15265161.2015.1062177, PMID: 26305757 PMC5234696

[37] Eder MM Carter-Edwards L Hurd TC Rumala BB Wallerstein N. A logic model for community engagement within the clinical and translational science awards consortium: can we measure what we model? Acad Med J Assoc Am Med Coll. (2013) 88:1430–6. doi: 10.1097/ACM.0b013e31829b54ae, PMID: 23752038 PMC3784628

[38] Komro KA Flay BR Biglan A Wagenaar AC. Research design issues for evaluating complex multicomponent interventions in neighborhoods and communities. Transl Behav Med. (2016) 6:153–9. doi: 10.1007/s13142-015-0358-4, PMID: 27012263 PMC4807196

[39] Trochim W Kane C Graham MJ Pincus HA. Evaluating translational research: a process marker model. Clin Transl Sci. (2011) 4:153–62. doi: 10.1111/j.1752-8062.2011.00291.x, PMID: 21707944 PMC3125608

[40] Hassmiller Lich K Frerichs L Fishbein D Bobashev G Pentz MA. Translating research into prevention of high-risk behaviors in the presence of complex systems: definitions and systems frameworks. Transl Behav Med. (2016) 6:17–31. doi: 10.1007/s13142-016-0390-z, PMID: 27012250 PMC4807191

[41] Glasgow RE Emmons KM. How can we increase translation of research into practice? Types of evidence needed. Annu Rev Public Health. (2007) 28:413–33. doi: 10.1146/annurev.publhealth.28.021406.144145 17150029

[42] Cross JE Pickering K Hickey M. Community-based participatory research, ethics, and institutional review boards: untying a Gordian knot. Crit Sociol. (2015) 41:1007–26. doi: 10.1177/0896920513512696

[43] Woolf SH. The meaning of translational research and why it matters. J Am Med Assoc. (2008) 299:211–3. doi: 10.1001/jama.2007.26, PMID: 18182604

[44] Chen SC Kim SY. A framework for analysis of research risks and benefits to participants in standard of care pragmatic clinical trials. Clin Trials. (2016) 13:605–11. doi: 10.1177/1740774516656945 27365010 PMC5133165

[45] Colemany C. Risk-benefit analysis In: Laurie G Dove E Ganguli-Mitra A McMillan C Postan E Sethi N, editors. The Cambridge handbook of health research regulation. Cambridge: Cambridge University Press (2021). 130–8.

[46] Shore N Drew E Brazauskas R Seifer SD. Relationships between community-based processes for research ethics review and institution-based IRBs: a national study. J Empir Res Hum Res Ethics. (2011) 6:13–21. doi: 10.1525/jer.2011.6.2.13, PMID: 21680973

[47] Michener L Cook J Ahmed SM Yonas MA Coyne-Beasley T Aguilar-Gaxiola S. Aligning the goals of community-engaged research: why and how academic health centers can successfully engage with communities to improve health. Acad Med J Assoc Am Med Coll. (2012) 87:285–91. doi: 10.1097/ACM.0b013e3182441680, PMID: 22373619 PMC3292771

[48] Brown P Morello-Frosch R Brody JG Altman RG Rudel RA Senier L. Institutional review board challenges related to community-based participatory research on human exposure to environmental toxins: a case study. Environ Health Glob Access Sci Sour. (2010) 9:39. doi: 10.1186/1476-069X-9-39, PMID: 20637068 PMC2914003

[49] Wolf LE Walden JF Lo B. Human subjects issues and IRB review in practice-based research. Ann Fam Med. (2005) 3:S30–7. doi: 10.1370/afm.302, PMID: 15928216 PMC1466958

[50] Yawn BP Graham DG Bertram SL Kurland MJ Dietrich AJ Wollan PC. Practice-based research network studies and institutional review boards: two new issues. J Am Board Fam Med. (2009) 22:453–60. doi: 10.3122/jabfm.2009.04.080168, PMID: 19587261

[51] Schräg ZM. Ethical Imperialism: Institutional Review Boards and the Social Sciences, 1965–2009. Baltimore, MD: Johns Hopkins University Press (2010).

[52] Wang C Burris MA. Photovoice: concept, methodology, and use for participatory needs assessment. Health Educ Behav. (1997) 24:369–87. doi: 10.1177/109019819702400309, PMID: 9158980

[53] McDonald LE Capous-Desyllas M. Navigating ethical issues in Photovoice: balancing the principles of community-based participatory research ethics with institutional review board requirements. J Empir Res Hum Res Ethics. (2021) 16:364–73. doi: 10.1177/15562646211032777, PMID: 34255580

[54] Lidz CW Pivovarova E Appelbaum P Stiles DF Murray A Klitzman RL. Reliance agreements and single IRB review of multisite research: concerns of IRB members and staff. AJOB Empir Bioeth. (2018) 9:164–72. doi: 10.1080/23294515.2018.1510437, PMID: 30285561 PMC6309766

[55] Engage for Equity. Available at: https://cpr.unm.edu/research-projects/cbpr-project/cbpr-e2.html (Accessed December 2, 2022).

[56] Kassis SB White SA Myers L Trudeau C Bierer BE. Advancing health literacy in clinical research: clear communications for every participant. NAM Perspect. (2019) 2019:1–7. doi: 10.31478/201910c, PMID: 34532669 PMC8406567

[57] Wilkins CH Alberti PM. Shifting academic health centers from a culture of community service to community engagement and integration. Acad Med J Assoc Am Med Coll. (2019) 94:763–7. doi: 10.1097/ACM.0000000000002711, PMID: 30893063 PMC6538435

[58] Fair M Johnson SB Fluker CJ Carkuff-Corey K. Health Equity in Academic Medicine: Recommendations from an AAMC Community Roundtable. Washington, DC: Association of American Medical Colleges (2021).

[59] Cargill SS DeBruin D Eder M Heitman E Kaberry JM McCormick JB. Community-engaged research ethics review: exploring flexibility in Federal Regulations. IRB. (2016) 38:11–9. PMID: 27301168 PMC4997782

[60] Lachance L Coombe CM Brush BL Lee SD Jensen M Taffe B. Understanding the benefit-cost relationship in long-standing community-based participatory research (CBPR) partnerships: findings from the measurement approaches to partnership success (MAPS) study. J Appl Behav Sci. (2022) 58:513–36. doi: 10.1177/0021886320972193, PMID: 36016649 PMC9398184

[61] Unertl KM Fair AM Favours JS Dolor RJ Smoot D Wilkins CH. Clinicians' perspectives on and interest in participating in a clinical data research network across the southeastern United States. BMC Health Serv Res. (2018) 18:568. doi: 10.1186/s12913-018-3399-9, PMID: 30029660 PMC6053753

[62] Graber MA Hartz A James P Nugent A Green MD. An alternative method of determining standard of care in alleged cases of malpractice. J Am Board Fam Pract. (2005) 18:453–8. doi: 10.3122/jabfm.18.6.453, PMID: 16322408

[63] Moffett P Moore G. The standard of care: legal history and definitions: the bad and good news. West J Emerg Med. (2011) 12:109–12. PMID: 21691483 PMC3088386

[64] Fischer MJ Go AS Lora CM Ackerson L Cohan J Kusek JW. CKD in Hispanics: baseline characteristics from the CRIC (chronic renal insufficiency cohort) and Hispanic-CRIC studies. Am J Kidney Dis. (2011) 58:214–27. doi: 10.1053/j.ajkd.2011.05.010, PMID: 21705121 PMC3577064

[65] Lora CM Ricardo AC Brecklin CS Fischer MJ Rosman Carmona E. Recruitment of Hispanics into an observational study of chronic kidney disease: the Hispanic chronic renal insufficiency cohort study experience. Contemp Clin Trials. (2012) 33:1238–44. doi: 10.1016/j.cct.2012.07.012, PMID: 22841929 PMC3594841

[66] Lynch HF Nicholls S Meyer MN Taylor HA. Consortium to advance effective research ethics oversight (AEREO). Of parachutes and participant protection: moving beyond quality to advance effective research ethics oversight. J Empir Res Hum Res Ethics. (2019) 14:190–6. doi: 10.1177/1556264618812625. Epub 2018 Dec 12, PMID: 30541368

[67] Anderson EE Hurley EA Serpico K Johnson A Rowe J Singleton M. Engaging key stakeholders to overcome barriers to studying the quality of research ethics oversight. Res Ethics. (2023) 19:62–77. doi: 10.1177/17470161221138028

[68] Tackett S Sugarman J Ng CJ Kamarulzaman A Ali J. Developing a competency framework for health research ethics education and training. J Med Ethics. (2022) 48:391–6. doi: 10.1136/medethics-2021-107237 33811112 PMC8486875

[69] Nicholls SG Hayes TP Brehaut JC McDonald M Weijer C Saginur R. A scoping review of empirical research relating to quality and effectiveness of research ethics review. PLoS ONE. (2015) 10:e0133639. doi: 10.1371/journal.pone.0133639, PMID: 26225553 PMC4520456

[70] Paasche-Orlow MK Brancati FL Taylor HA Jain S Pandit A Wolf MS. Readability of consent form templates: a second look. IRB: Ethics Hum Res. (2013) 35:12–9. PMID: 23926857

[71] Kahn AP Mastroianni AC Sugarman J. Beyond Consent: Seeking Justice in Research. New York: Oxford University Press (1998).

[72] Morgenstern J Hegele RA Nisker J. Simple genetics language as source of miscommunication between genetics researchers and potential research participants in informed consent documents. Public Underst Sci. (2015) 24:751–66. doi: 10.1177/0963662514528439, PMID: 24751688

[73] Emanuel EJ Boyle CW. Assessment of length and readability of informed consent documents for COVID-19 vaccine trials. JAMA Netw Open. (2021) 4:e2110843. doi: 10.1001/jamanetworkopen.2021.10843, PMID: 33909052 PMC8082317

[74] Fullerton SM Anderson EE Cowan K Malen RC Brugge D. Awareness of Federal Regulatory Mechanisms Relevant to community-engaged research: survey of health disparities-oriented NIH-funded investigators. J Empir Res Hum Res Ethics. (2015) 10:13–21. doi: 10.1177/1556264614561964, PMID: 25742662 PMC4413468

[75] Hayes GJ Hayes SC Dykstra T. A survey of university institutional review boards: characteristics, policies, and procedures. IRB: Ethics Hum Res. (1995) 17:1–6. doi: 10.2307/3563605 11654267

[76] Flicker S Travers R Guta A McDonald S Meagher A. Ethical dilemmas in community-based participatory research: recommendations for institutional review boards. J Urban Health. (2007) 84:478–93. doi: 10.1007/s11524-007-9165-7, PMID: 17436114 PMC2219570

[77] Kahnawake Schools Diabetes Prevention Project Code of Research Ethics (2007). Available at: https://www.ksdpp.org/uploads/1/3/6/4/136499863/appendix-a-ksdpp_code_of_research_ethics2007.pdf 10093242

[78] Kuhn NS Parker M Lefthand-Begay C. Indigenous research ethics requirements: an examination of six tribal institutional review board applications and processes in the United States. J Empir Res Hum Res Ethics: JERHRE. (2020) 15:279–91. doi: 10.1177/1556264620912103, PMID: 32233729

[79] Mills CW. The Sociological Imagination. New York, NY: Oxford University Press (1959).

[80] Habermas J. Knowledge and Human Interests. Boston, MA: Beacon Press (1971).

[81] Association of American Medical Colleges, Principles of Trustworthiness (2021). Available at: https://www.aamchealthjustice.org/media/271/download?attachment (Accessed April 24, 2022).

